# Selenium and cancer prevention

**DOI:** 10.1186/1897-4287-10-S4-A1

**Published:** 2012-12-10

**Authors:** Margaret Rayman

**Affiliations:** 1 Department of Nutrition and Metabolism, Faculty of Health and Medical Sciences, University of Surrey, UK

## 

The essential trace mineral, selenium, has been the focus of an increasing number of research studies since the 1996 report that it decreased the risk of some common cancers. Prospective studies have generally shown some benefit of higher selenium status on the risk of prostate, lung, colorectal, and bladder cancers but trials have had mixed findings, likely highlighting the fact that supplementation will only confer benefit if intake of a nutrient is inadequate. There is evidence for the involvement of a plethora of mechanisms in the anti-cancer effects of selenium including protection from oxidative damage to DNA and stimulation of DNA repair mechanisms, which may be particularly relevant to genetic cancers. Research effort has focussed on low molecular weight selenium species and selenoproteins as cancer preventive agents. The importance of selenoproteins has been demonstrated by the fact that single nucleotide polymorphisms in selenoprotein genes affect the risk of a number of cancers as does methylation of the promoter region of the GPx3 gene which inhibits expression of this protective selenoprotein. Selenium intake, and therefore status, varies tremendously across the world demonstrating both deficiency and toxicity. Trial evidence has suggested that supplementing those who already have adequate selenium intake and maximal selenoprotein activity/concentration with additional selenium may increase the risk of alopecia, dermatitis and type-2 diabetes, reminding us that selenium was first known as a toxic element. The crucial factor that needs to be emphasized with regard to the health and anti-cancer effects of selenium is the inextricable U-shaped link with status: while additional selenium intake may well benefit people with low status, those of adequate-to-high status may be affected adversely and should not take selenium supplements.

